# Light‐Activated Pharmacological Tools for Exploring the Cholinergic System

**DOI:** 10.1002/med.22108

**Published:** 2025-03-23

**Authors:** Alessio Colleoni, Giulia Galli, Clelia Dallanoce, Marco De Amici, Pau Gorostiza, Carlo Matera

**Affiliations:** ^1^ Section of Medicinal Chemistry “Pietro Pratesi”, Department of Pharmaceutical Sciences University of Milan Milan Italy; ^2^ Department of Chemistry, Biology, and Biotechnology University of Perugia Perugia Italy; ^3^ Catalan Institution for Research and Advanced Studies (ICREA) Barcelona Spain; ^4^ Institute for Bioengineering of Catalonia (IBEC), The Barcelona Institute for Science and Technology Barcelona Spain; ^5^ Biomedical Research Networking Center in Bioengineering, Biomaterials, and Nanomedicine (CIBER‐BBN) Madrid Spain

**Keywords:** muscarinic acetylcholine receptors, nicotinic acetylcholine receptors, photopharmacology, photoswitch, uncaging

## Abstract

Cholinergic transmission plays a critical role in both the central and peripheral nervous systems, affecting processes such as learning, memory, and inflammation. Conventional cholinergic drugs generally suffer from poor selectivity and temporal precision, leading to undesired effects and limited therapeutic efficacy. Photopharmacology aims to overcome the limitations of traditional drugs using photocleavable or photoswitchable ligands and spatiotemporal patterns of illumination. Spanning from muscarinic and nicotinic modulators to cholinesterase inhibitors, this review explores the development and application of light‐activated compounds as tools for unraveling the role of cholinergic signaling in both physiological and pathological contexts, while also paving the way for innovative phototherapeutic approaches.

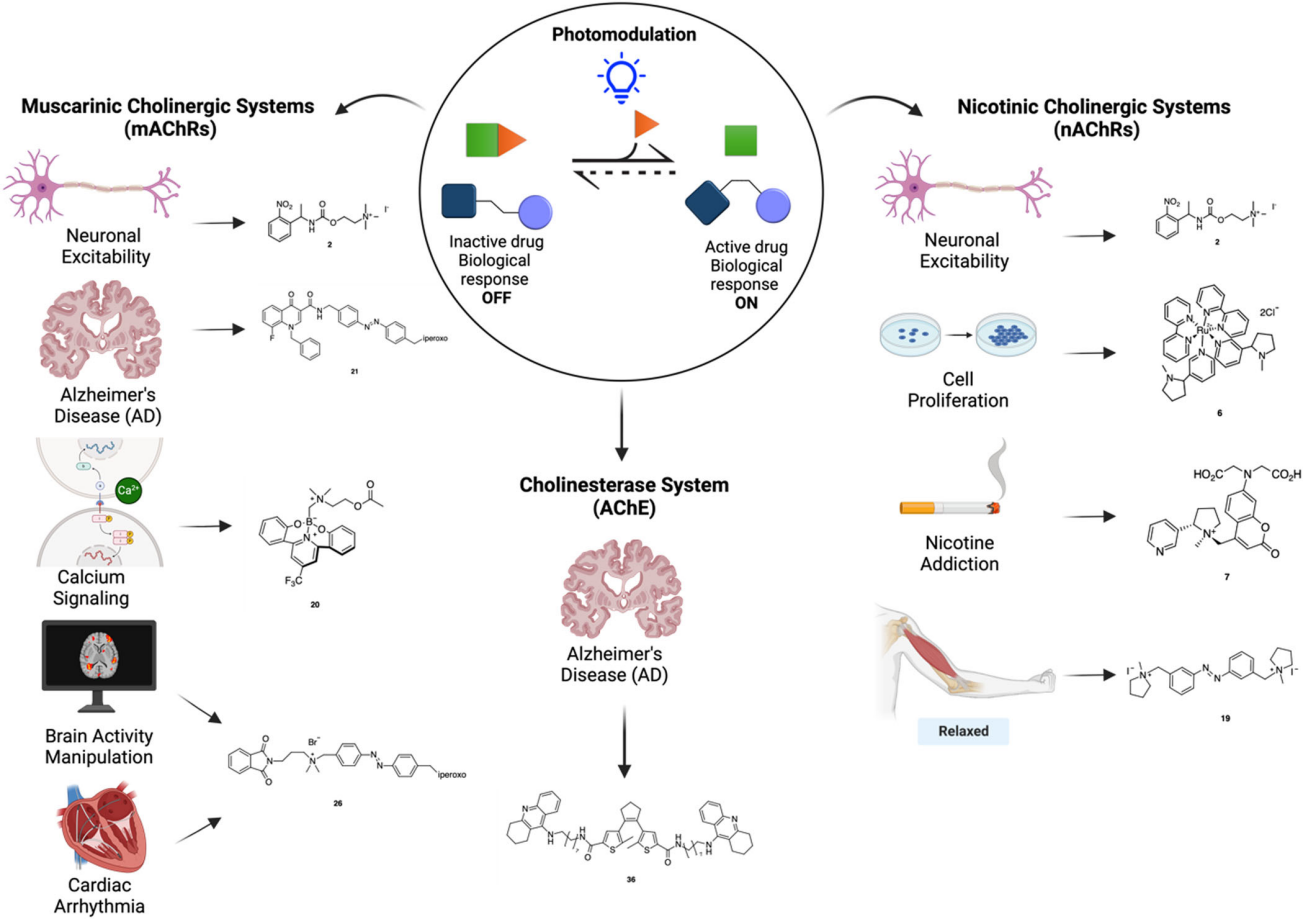

## 1 Receptors and Enzymes Mediating Cholinergic Signaling

Cholinergic transmission plays a key role in several physiological processes and pathological conditions. Acetylcholine (ACh) was the first substance to be identified as a neurotransmitter and since then many studies have focused on the understanding of its mechanisms and functions. The biosynthesis of ACh starts with the decarboxylation of serin catalyzed by serine decarboxylase, providing ethanolamine, which is then methylated to choline, a quaternary ammonium salt, by the enzyme *N*‐methyltransferase. Choline is then absorbed in the cytoplasm of neurons where it is converted into acetylcholine by the enzyme choline acetyl transferase, which uses as substrate acetyl‐CoA produced in mitochondria [1]. Once synthesized, the vesicular acetylcholine transporter loads ACh into synaptic vesicles, which are docked near presynaptic membranes. When an action potential arrives at the presynaptic terminal, it triggers the opening of voltage‐gated calcium channels. The increase of Ca^2+^ concentration causes the fusion of the synaptic vesicles containing ACh with the presynaptic membrane and the neurotransmitter release. ACh exerts its effects both in the central nervous system (CNS) and in the peripheral nervous system (PNS) through two families of acetylcholine receptors (AChRs)—the nicotinic AChRs (nAChRs) and the muscarinic AChRs (mAChRs). In addition to its action as a typical neurotransmitter when released by cholinergic neuronal terminals, ACh from non‐neuronal tissues is involved in cell‐to‐cell communication, and controls essential functions such as cell proliferation, adhesion, migration, secretion, survival, and apoptosis, in an autocrine, paracrine or juxtacrine manner [2]. Together with that released by vagal nerve endings, ACh also contributes to the cholinergic control of inflammation [3]. To keep the synapse ready for the next transmission event, the action of ACh is rapidly terminated by enzymatic hydrolysis, mainly catalyzed by acetylcholinesterase (AChE) and secondly at a slower rate by butyrylcholinesterase (BuChE), resulting in choline, which is transported back into the presynaptic neuron by a choline transporter and re‐used for the synthesis of further ACh.

nAChRs are pentameric ligand‐gated ion channels that are present in both the PNS (at the skeletal neuromuscular junction and in the autonomic nervous system) and the CNS. Neuronal nAChRs consist of nine α (α_2_–α_10_) and three β (β_2_–β_4_) subunits arranged as homo‐ or heteropentamers [4]. The homomeric (α_7_ or α_9_) or heteromeric (α_2_–α_6_ with β_2_–β_4_) assembly of five subunits generates many distinctive subtypes that share a common basic structure [4]. The most widely expressed neuronal subtypes in the brain are heteromeric α_4_β_2_* (the asterisk indicates that other subunits may be co‐assembled) and homomeric α_7_ receptors, whereas α_3_β_4_* is the most widely expressed subtype in the PNS [5]. In the brain, nAChRs are involved in several functions including learning and memory, arousal, reward, motor control, analgesia. nAChRs are not only permeable to monovalent Na^+^ and K^+^ ions, but also to Ca^2+^ ions. The ability of nAChRs to alter intracellular calcium levels leads to activation of different downstream intracellular pathways that contribute to regulate neuronal signaling and plasticity [6]. Due to their involvement in regulating cognitive processes, changes in their number and function are associated with various pathological conditions such as anxiety, depression, Alzheimer's and Parkinson's diseases, among others [7]. nAChRs are also expressed in epithelial cells, immune cells, and mitochondria. These receptors, that are termed non‐neuronal nAChRs, have been found to participate in inflammation, maintenance of epithelial cells homeostasis, and epithelial tumor progression. In mitochondria, they modulate initial events of apoptosis through the cascade of signaling kinases [8].

Overall, the functional properties of each nAChR subtype are unique but overlap sufficiently to make them very difficult to distinguish using conventional pharmacological agents, especially when the subtypes have subunits in common or contain different subunits with a high degree of homology [9, 10]. Although many promising ligands selectively active on nAChRs have been identified or developed over the years, their clinical application has failed or proceeded very slowly. Most clinical studies have been interrupted either because the beneficial effects were insufficient, or side effects were intolerable. Furthermore, as the nicotinic system generally seems to act as a fine tuner or modulator of very complex and articulated physiological processes rather than behaving as an on/off switch, the effects of nicotinic drug candidates are generally difficult to dissect. Among the nicotinic agonists/partial agonists, the only drug that has been marketed, more than 15 years ago, was varenicline, indicated for smoking cessation therapy. On the whole, despite the relevant therapeutic potential of nicotinic candidate drugs, so far their clinical translation has always proved difficult and, as a consequence, the interest of pharmaceutical companies in this target has progressively declined [11]. The possibility to control nAChRs with high spatiotemporal precision would open up new opportunities for studying brain function and behavior, particularly in the context of CNS pathologic conditions like Alzheimer's disease (AD), schizophrenia, and nicotine addiction.

mAChRs belong to the superfamily of G protein‐coupled receptors (GPCRs) and comprise five distinct subtypes, M_1_–M_5_, that mainly differ in their expression pattern, G protein‐specificity, and molecular structure. M_1_, M_3_, and M_5_ receptors couple to G_q/11_ proteins increasing the formation of inositol‐1,4,5‐trisphosphate, activating phospholipase C, mobilizing intracellular calcium and increasing cAMP levels. On the other hand, M_2_ and M_4_ receptors exert their effects by binding primarily to G_i/o_ proteins, inhibiting the activity of adenylyl cyclase and decreasing cAMP formation. The overall structures are highly similar, with the greatest structural homology within the ACh binding site (i.e., the orthosteric binding site), which highlights why subtype‐selective targeting of muscarinic receptors has never been achieved so far with pure orthosteric agonists. However, some extracellular transmembrane domains and protein loops of the receptors are characterized by lower degrees of sequence homology, and in these regions the common allosteric binding site of mAChRs has been identified. This gives the opportunity to exploit this less conserved recognition site for a putative subtype selective targeting of mAChRs [12]. All mAChR subtypes are widely expressed in the CNS, where they mediate sensory, motor, cognitive, and vegetative functions [13], and in the PNS, where they regulate the parasympathetic actions of ACh [14]. Since mAChRs are involved in many circuits, they are regarded as highly relevant therapeutic targets. For instance, M_1_ receptors are highly expressed in the cerebral cortex, hippocampus, and striatum. This is consistent with their role in learning and memory, and selective M_1_ orthosteric ligands have long been investigated to improve cognitive decline in AD [15]. M_2_ receptors mediate vagal modulation of heart tissue, whereas M_3_ receptors are located mainly on glandular and respiratory tissues, regulating glandular mucus secretion and bronchoconstriction, respectively. Therapeutic exploitation of mAChR ligands is currently limited to muscarinic antagonists. As an example, blocking of M_3_ mAChR responses in the PNS is an option in use to treat disorders of smooth muscle function, such as irritable bowel syndrome, urinary urge incontinence, and chronic obstructive pulmonary disease (COPD) [16]. Irritable bowel syndrome is usually treated with loperamide (an opioid receptor agonist) and cisapride (a 5‐HT_4_ receptor agonist), but muscarinic antagonists are frequently used in association to reduce abdominal pain and colonic hypermotility [16, 17]. Urinary urge incontinence can be treated with oxybutynin, another non‐selective mAChR antagonist [18]. Moreover, ipratropium is commonly used in combination with β_2_ agonists to inhibit bronchoconstriction in COPD patients [19]. However, all these drugs show the typical adverse effects of anti‐cholinergic medications, such as dry‐mouth, blurred vision, constipation, and tachycardia [16]. Accordingly, developing selective drugs for these receptor subtypes would be a groundbreaking advancement, enhancing the overall therapeutic effectiveness of muscarinic ligands while concomitantly minimizing the undesirable side effects of current drug substances. This could lead to more precise and safer treatments for a variety of conditions, ultimately improving patient outcomes and enabling personalized medicine.

AChE and BuChE are serine hydrolases that split the neurotransmitter ACh and terminate its action. Regarding the tissue distribution of the two enzymes in the body, AChE is mainly involved in neuronal signals, both in the CNS (i.e., at the cholinergic neurons) and in the PNS (i.e., at the neuromuscular junction) [20]. BuChE is distributed in different districts of the body, as it has been found in the brain, plasma, heart, liver, and lungs [20]. AChE plays the key role in ending cholinergic neurotransmission. The molecular structure of the enzyme presents different key sites: catalytic active site (CAS), peripheral aromatic site (PAS), oxyanion hole (OAH), Ω‐loop (OML), and acyl binding site (ABS). Computational investigations have proved that the primary sites for binding are CAS and PAS [21]. AChE has been investigated for a long time as therapeutic target, following the discovery that it is involved in neurodegenerative diseases. Nowadays AChE inhibitors are the most common class of drugs prescribed to relief AD symptoms, including donepezil, galantamine, and rivastigmine. Although these compounds interact differently with the enzyme, they all have something in common, i.e. a limited efficacy and several dose‐associated side effects, like nausea, loss of appetite, insomnia, stomach cramps, muscle weakness; in addition, rivastigmine at higher doses can also cause chest pain and irregular heartbeat [22].

Since patients suffering from AD usually show comorbidities due to the illness, these side effects can heavily affect their quality of life. Although the majority of studies for the treatment of AD focus on the inhibition of AChE, recently a new approach has been emerging, which consists in developing inhibitors of BuChE, that apparently can compensate for the decreased function of AChE [23, 24]. In 2007 it was reported that an increase of ACh was observed when BuChE was inhibited, which basically means that when AChE activity decreases, BuChE activity increases in the brain [25]. In addition, several studies demonstrated that BuChE is involved in the formation of amyloid‐beta plaques, recognized as one of the most relevant drivers of the AD pathogenesis and progression [26]. On one hand, this suggests that BuChE activity stimulates the maturation of the amyloid‐beta plaques, on the other, the connection between BuChE and the plaques allows to consider BuChE a diagnostic target for AD [20]. However, despite its involvement in several physiological processes, there are no BuChE‐selective inhibitors approved so far. Overall, developing precise methods to control cholinesterase activity could significantly enhance treatment for neurodegenerative diseases. For instance, by targeting specific brain regions, such as the hippocampus or cortex, these inhibitors could improve cognitive function more effectively. Additionally, responsive technologies could activate the drug only when needed, and personalized approaches based on genetic profiles or imaging could optimize a given treatment, improving overall efficacy as well as patient comfort.

As underlined in the previous paragraphs, ACh assumes a crucial role in several patho‐ physiological pathways. This multifaceted engagement is achieved through the activation of nicotinic and muscarinic receptors, and its clearance from the synaptic cleft by means of cholinesterases. Over the past few decades, there has been substantial and comprehensive investigation into the cholinergic transmission machinery. The primary objective of this inquiry has contributed to the elucidation of several intricate mechanisms governing physiological responses. Additionally, this pursuit has sought to discern the pathogenesis of disorders impacting on both the CNS and the PNS, thereby fostering the identification of promising cholinergic ligands. A fair number of pharmacological agents targeting the cholinergic transmission has been developed, but their poor subtype‐ or tissue‐selectivity generally causes adverse effects. Moreover, the temporal precision of these drugs is strictly dependent on their pharmacokinetics. The lack of spatiotemporal precision prevented researchers from designing effective therapies and fully understanding the role of cholinergic transmission in both physiological and pathological pathways. Overall, the limitations of traditional pharmacology in the cholinergic as well as in other research fields have motivated the development of advanced pharmacological techniques for higher‐resolution studies, among them the photochemical approaches aimed to generate biologically active derivatives.

## 2 The Photopharmacological Approach

The use of photochemistry has allowed to achieve quite relevant results, in terms of both basic research and applications, in different research fields, ranging from synthetic organic to supramolecular chemistry, from energy storage to water splitting and CO_2_ reduction, from photopolymerization and photocuring to photobiology and phototherapy [27]. Among the techniques and strategies linked with potential therapeutic intervention [28], the employment of light promotes a dynamic control of drug activity in a highly resolved spatiotemporal manner. Light‐mediated drug action may be, therefore, beneficial in overcoming the two main issues of traditional pharmacotherapy, i.e., the poor selectivity of drug action, responsible for unwanted or toxic side effects, and the occurrence of drug resistance, mostly regarding the administration of antimicrobials—antibiotics, antivirals, antifungals, and antiparasitics—that become increasingly ineffective.

Photopharmacology [29], a combination of “photochemistry” and “pharmacology”, emerged in the 1960s and has rapidly grown over the past 20 years. It relies on the use of synthetic, rationally designed, bioactive compounds incorporating a phototunable moiety. Light irradiation is thus able to induce a structural modification of the photopharmacological agent, which allows to regulate its biological action on a selected native target using temporal and spatial patterns of illumination. On the way to attain more efficacious therapeutic modalities, the potential of photopharmacology has taken advantage of the achievements of other established light‐based biomedical or investigational technologies, such as photodynamic therapy (PDT) and optogenetics.

PDT encompasses light‐based processes that offer non‐conventional therapeutic options for a variety of solid tumors, even as components of combination treatments, since PDT is usually non‐cross‐resistant with other cancer treatments [30]. This clinical therapy involves the application of a photosensitive compound, known as photosensitizer (PS), which accumulates in diseased tissues. When exposed to light of a specific wavelength, the PS absorbs the light and triggers activation processes that lead to the targeted destruction of abnormal cells. These photocytotoxic reactions are confined to the area where the photosensitizer and the light are present, ensuring selective destruction of the diseased tissue [31]. The improvement of technological devices used in PDT, such as lasers, cheap and compact light emitting diodes (LEDs), and two‐photon based systems for a spatially precise activation of PSs in tissues, aimed at a cost‐effective delivery of light with a highly regulated dose and wavelength, thus reducing the poor depth of penetration and the cellular photodamage of UV radiation. The progress in the instrumentation developed for PDT also contributes to ameliorating the technological solutions to be adopted to improve safety and selectivity in photopharmacological applications.

On the other hand, optogenetics harnesses the power of light to regulate the activity of genetically encoded ion channels, receptors, transporters, and enzymes, derived from excitatory or inhibitory opsins, and even of chimeric GPCRs, such as OptoXRs, combining photo‐responsive rhodopsin domains with the intracellular sequences of the target GPCR [32]. Intracellular signaling cascades have thus been modulated by engineering photo‐activatable cell‐surfaced GPCRs, among them adrenergic, serotoninergic, dopaminergic, adenosine, glutamate (metabotropic), and μ‐opioid receptors, able to generate the same signaling cascade as the endogenous receptors, whereas they are triggered with a spatiotemporal precision that is not feasible with traditional pharmacology [32]. Optogenetics made substantial contributions to clarify the roles of specific neuronal populations of various neurotransmitter systems or to study neuronal circuits. Besides providing a powerful tool for in‐depth investigation of neuronal mechanisms, optogenetics reveals an interesting potential for specific clinical applications. As an example, for the treatment of degenerative retinal diseases, inherited genetic modifications causing photoreceptor degeneration may be corrected by using gene‐replacement therapy that uses viral vectors to replace or introduce mutant genes, thus slowing down the disease progression or restoring visual function in animal models of retinal degeneration and sometimes even in human patients [33]. In principle, if gene‐replacement therapy in vision restoration may start even before visual function is lost, the ensuing clinical impact suffers from the restriction of requiring tailored genetic manipulations of biological targets, thus hindering the translational path, and from a predictable lower efficacy in those cases of more advanced retinal degenerative diseases [32, 34].

The advent of photopharmacology has enabled the strategic incorporation of light‐responsive moieties into the molecular framework of biologically active compounds, generally without requiring any ad hoc genetic engineering modification of the target protein structure. Upon irradiation, these added moieties modify the overall structural features of their related bioactive compound, ideally “switching” the ligand from an “off” to an “on” state, only in illuminated regions, and remaining safely inactive elsewhere. These structural changes involve different recognition modes of the biological target, thus leading to relevant variations of the pharmacodynamic properties that promote a light‐activated response [35, 36]. It is possible to identify two types of photopharmacology depending on whether the activation of the light‐regulated bioactive compound is permanent or not: “irreversible” and “reversible” photopharmacology, respectively [37].

Irreversible photopharmacology makes use of *photocleavable* protecting groups (PPGs or “cages”) that are covalently bound to a given compound in a way that transiently deactivates its biological properties. With the cleavage of PPGs by light irradiation (“uncaging”), the absorbed energy is translated into the restoration of biological activity, switching on (or off) the targeted process in a spatiotemporally regulated manner [36]. Similarly to prodrugs, photocleavable ligands are compounds that are endowed with a photoremovable caging group which is released upon irradiation, thus unleashing the biologically active moiety. This strategy has some undeniable advantages: in principle it can potentially be applied to most of biologically active ligands and offers a wide variety of options in the choice of the photoremovable group depending on both the functional group where the cage will be attached and the wavelength of light that one envisages to use for the photolysis [38]. However, the application of photocleavable ligands suffers from the lack of reversibility, potential generation of byproducts with unwanted biological activities, and low efficacy of photo‐uncaging, and is limited spatially by the diffusion of the released bioactive compound [39].

On the other hand, *photoswitchable* ligands are photoisomerizable bioactive compounds that enable to control reversibly the activity of biological targets with light. These compounds incorporate a photochromic moiety, often an azobenzene or more generally an azoarene, that serves as conformational switch. Under proper illumination the switch triggers a change in the geometry of the pharmacophore which translates into a change in the affinity and/or efficacy of the ligand [39]. Unlike photocleavable ligands, these molecules offer the great advantage of enabling reversible spatiotemporal control of biological targets. However, the applicability of this strategy is more limited: indeed, it is not always possible to incorporate a molecular photoswitch into the structure of a biologically active derivative while retaining the activity profile in one of the two possible conformational states. Moreover, the molecular photoswitches that have been the most successful in photopharmacology—namely azobenzenes and, more broadly, azoarenes—generally exhibit suboptimal photochemical properties and often require short‐wavelength light to trigger isomerization.

**Figure 1:**
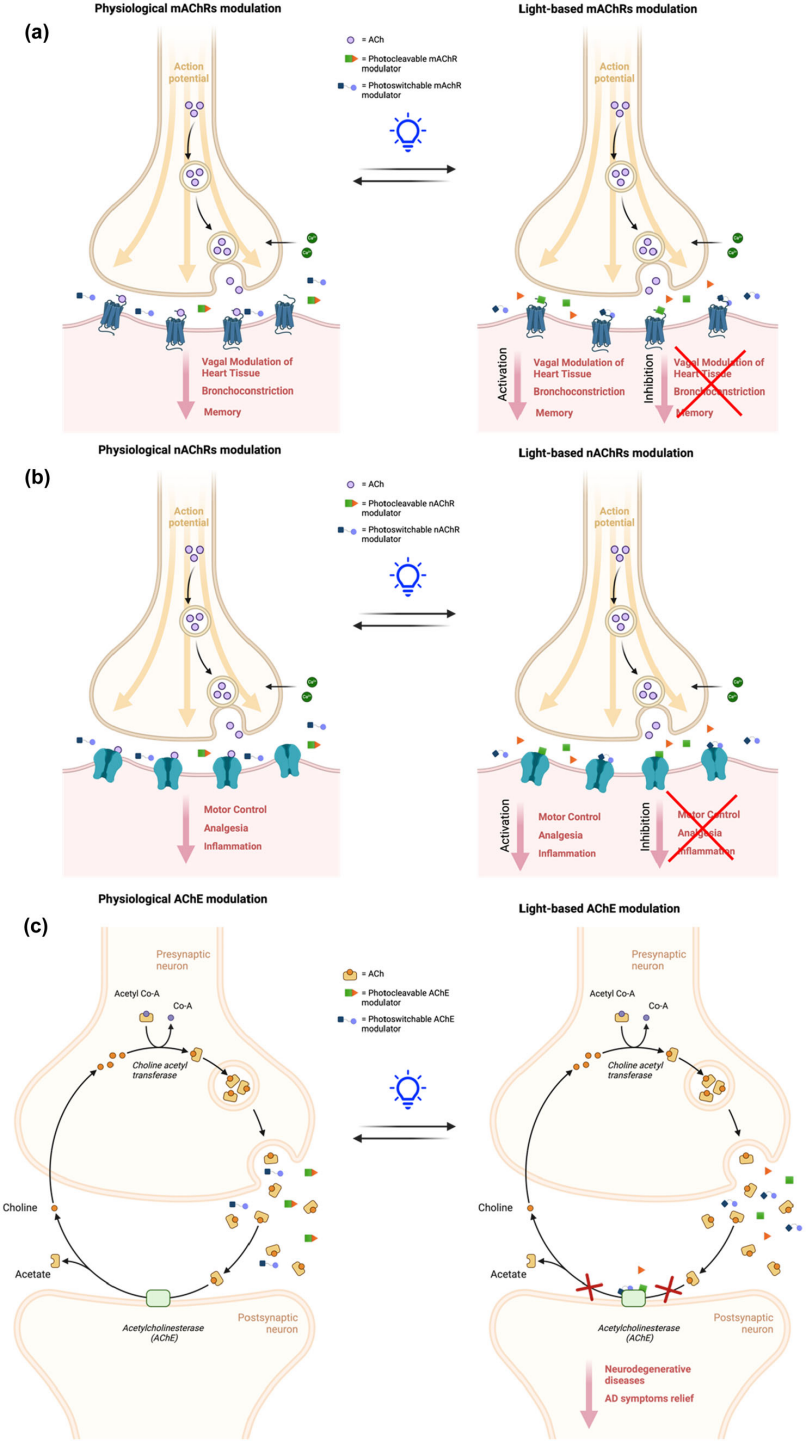
Representative sketch of cholinergic signaling under physiological conditions versus light‐based modulation. Comparison of natural cholinergic transmission (left) and light‐controlled modulation (right) at nicotinic (a) and muscarinic (b) acetylcholine receptors, as well as acetylcholinesterase (c), highlighting their functional effects. [Color figure can be viewed at wileyonlinelibrary.com]

Photopharmacology, therefore, offers a new pathway towards precision medicine and provides new valuable research tools to dissect the contribution of nAChRs, mAChRs, and cholinesterases to the cholinergic transmission (Figure 1). In this review we summarize and discuss the most relevant achievements in cholinergic photopharmacology and the applications of light‐responsive ligands to the investigation of cholinergic transmission (see also the **Table of Compounds** at the end of the manuscript).

**Table of Compounds**

**Table:** 

Number	Compound	Biological activity	Wavelength of activation (nm)	Reference	
***Photocleavable cholinergic modulators***					
**1**	*N*‐(2‐Nitrobenzyl)‐ carbamoylcholine odide	Cholinergic agonist	300–355	Hess et al. (1986) [40]	
**2**	1‐(2‐Nitrophenyl)ethyl] carbamoylcholine iodide	Cholinergic agonist	300–355	Hess et al. (1986) [40]	
**3**	2‐Bromoethyl‐*N*‐(2‐nitrobenzyl)‐carbamate	Cholinergic agonist	300–355	Hess et al. (1989) [41]	
**4**	2‐Bromoethyl‐*N*‐(4‐carboxy‐2‐nitrobenzyl)‐carbamate	Cholinergic agonist	300–355	Hess et al. (1989) [41]	
**5**	*N*‐[(α‐Carboxy)2‐nitrobenzyl] carbamylcholine trifluoroacetate	Cholinergic agonist	300–355	Hess et al. (1989) [41]	
***Photocleavable nicotinic modulators***					
**6**	RuBi‐Nicotine	Nicotinic agonist	473 and 532	Etchenique et al. (2010) [42]	
**7**	PA‐Nic	Nicotinic agonist	200–400 (UV light)	Lavis et al. (2018) [43]	
**8**	DPNB‐ABT594	Nicotinic agonist	365	Ellis‐Davies et al. (2018) [44]	
***Photoswitchable cholinergic and cholinesterase antagonists***					
**9**	Azo‐PTA	Inhibitor of AChR/AChE	320 (*trans*)	Erlanger et al. (1969) [45]	
**10**	Azo‐Ph‐carbachol	Inhibitor of AChR/AChE	320 (*trans*)	Erlanger et al. (1969) [45]	
***Photoswitchable nicotinic modulator***					
**11**	BisQ	nAChR agonist/mAChR antagonist	330 (*trans*)	Erlanger, Wassermann et al. (1971) [46]	
**12**	QBr	nAChR agonist	380 (*trans*)	Erlanger, Wassermann et al. (1971) [47]	
**13**	MAACh	nAChR agonist	380 (*trans*)	Trauner et al. (2015) [48]	
**14**	MAHoCh	nAChR antagonist	380 (*trans*)	Trauner et al. (2015) [48]	
**15**	AzoCholine	α7 nAChR agonist	360 (*trans*) and 440 (*cis*)	Trauner et al. (2015) [49]	
**16**	d_9_‐AzoCholine	α7 nAChR agonist	455 (*trans*)	Li et al. (2024) [50]	
**17**	4FABTA	nAChR agonist	380 (*trans*) 550 (*cis*)	Ellis‐Davies et al. (2019) [51]	
**18**	Azocuroniums	nAChR modulators	335–340 (*trans*)	Rodrìguez‐Franco et al. (2020) [52]	
**19**	**m‐1a** (*meta*‐Azocuronium)	nAChR muscle‐type selective antagonist	335–340 (*trans*)	Rodrìguez‐Franco et al. (2020) [52]	
***Photocleavable muscarinic modulators***					
**20**	CagMe‐ACh	Muscarinic agonist	405	Ohmiya, Arai et al. (2023) [53]	
***Photoswitchable muscarinic modulators***					
**21**	BQCAAI	M1 mAChR agonist (*trans*) and antagonist (*cis*)	360 (*trans*) and 460 (*cis*)	Decker et al. (2017) [54]	
**22**	Photo‐iperoxo	mAChR agonist	365 (*trans*), 320 and 422 (*cis*)	Decker et al. (2019) [55]	
**23**	Bivalent photo‐iperoxo	mAChR agonist	365 (*trans*), 320 and 422 (*cis*)	Decker et al. (2019) [55]	
**24**	Fluorinated photo‐iperoxo	mAChR agonist	500 (*trans*) and 400 (*cis*)	Decker et al. (2019) [55]	
**25**	Fluorinated bivalent photo‐iperoxo	mAChR agonist	500 (*trans*) and 400 (*cis*)	Decker et al. (2019) [55]	
**26**	Phthalimide‐Azo‐Iperoxo (PAI)	M2 mAChR agonist	365 (*trans*) and 460 (*cis*)	Riefolo, Matera et al. (2019) [56]	
**27**	Cryptozepine 2	M1 mAChR antagonist	365 (trans) and 460 (cis)	Riefolo et al. (2021) [57]	
**28**	Cryptozepine 3	M1 mAChR antagonist	460‐500 (*trans*) and dark light (*cis*)	Riefolo et al. (2021) [57]	
**29**	photo‐BQCisA	M1 mAChR PAM	365	Gerwe et al. (2024) [58]	
**30**	photo‐BQCtrAns	M1 mAChR PAM	470	Gerwe et al. (2024) [58]	
***Photocleavable cholinesterase modulators***					
**31**	Choline 2‐Nitrobenzyl Ether A	AChE and BuChE inhibitor	351		Peng, Goeldner et al. (1996) [59]
**32**	Choline 2‐Nitrobenzyl Ether B	AChE and BuChE inhibitor	351		Peng, Goeldner et al. (1996) [59]
**33**	Choline 2‐Nitrobenzyl Ether C	AChE and BuChE inhibitor	351		Peng, Goeldner et al. (1996) [59]
**34**	Caged oxime 2	Reactivator of paraoxon‐inactivated AChE	365 and 420		Choi et al. (2022) [60]
**35**	Caged oxime 3	Reactivator of paraoxon‐inactivated AChE	365		Choi et al. (2022) [60]
***Photoswitchable cholinesterase modulators***					
**36**	DTE‐THA	AChE inhibitor	320 (closing) and > 420 (opening)	Decker et al. (2014) [61]	
**37**	AzoTHA	AChE inhibitor	350 (*trans*)	Trauner et al. (2014) [62]	
**38**	Rev‐BChE‐inh ‐9	BuChE inhibitor	365 (*trans*) and 400 (*cis*)	Decker, Maurice et al. (2022) [63]	
**39**	Rev‐BChE‐inh‐10	BuChE inhibitor	365 (*trans*) and 400 (*cis*)	Decker, Maurice et al. (2022) [63]	
**40**	1c	AChE and MAO‐B inhibitor	450–495 (*E*)	Altomare, Corelli et al. (2022) [64]	
**41**	1h	AChE inhibitor	334–340 (*E*)	Altomare, Corelli et al. (2023) [65]	

## 3 Nicotinic Acetylcholine Receptors

### 3.1 Photocleavable Ligands

**Figure 2:**
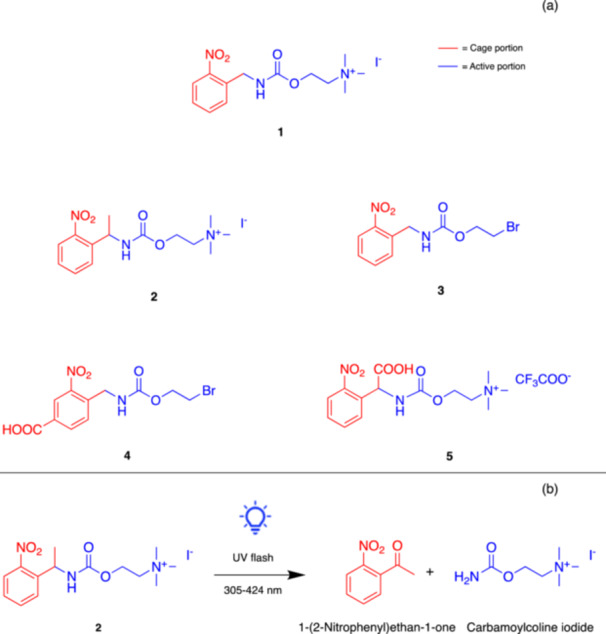
(a) First photocleavable cholinergic modulators reported in the literature. (b) Photolytic reaction of “cage I” (**2**) to release carbamoylcholine iodide. [Color figure can be viewed at wileyonlinelibrary.com]

To the best of our knowledge, the first report on the design, synthesis, and characterization of photocleavable nicotinic ligands was published in 1986 by Hess and coll [40]. They synthesized two compounds (**1** and **2**, Figure 2a) featuring a photoremovable 2‐nitrobenzyl moiety attached directly to the carbamate nitrogen of carbachol (carbamoylcholine), a non‐selective (nicotinic and muscarinic) cholinergic agonist. Carbachol could be released from these derivatives in response to laser light pulses at wavelengths between 300 and 355 nm, and activate, after photolysis, the nAChR, as determined by ^86^Rb^+^ flux measurements with membrane vesicles prepared from *Torpedo californica* and *Electrophorus electricus*. Instead, the two “caged” derivatives (non‐photolyzed) interacted only weakly with the ACh binding sites, as shown by competitive inhibition of ACh‐stimulated flux at high concentrations. Following these results, in 1989 Hess and coll. developed three other carbamate derivatives of carbachol (**3**, **4**, and **5**, Figure 2a) bearing the photolabile 2‐nitrobenzyl moiety [41]. Unlike the former set of derivatives, Compound **5** resulted completely inactive in the caged form.

Some of these caged carbachol derivatives found application in further studies involving nAChRs. In 1992 Görne‐Tschelnokow and coll. described the use of “cage I” (*N*‐[1‐(2‐nitrophenyl)ethyl] carbamoylcholine iodide, Compound **2**, Figure 2a,b) and “cage II” (*N*‐[(α‐carboxy)‐2‐nitrobenzyl] carbamylcholine trifluoroacetate, Compound **5**, Figure 2a) to investigate agonist binding and receptor conformational changes by Fourier transform infrared (FTIR) spectroscopy [66]. Upon UV flash activation of caged carbachol derivatives, characteristic changes in the IR absorbance spectrum were detected, indicating small but specific conformational changes probably involving a few amino acids only.

In another study, Hess et al. used caged carbachol *N*‐[(α‐carboxy)‐2‐nitrobenzyl] carbamylcholine trifluoroacetate **5** to determine the effect of a local anesthetic, procaine, on the apparent rate constants for both channel opening and closing of nAChRs. Nicotinic channels are blocked by cationic inhibitors, including local anesthetics, and ACh at high concentrations due to desensitization. The inhibitors may affect only the apparent rate constant for channel closing (k'_closing_), with compounds entering the receptor‐channel after it opens and blocking inorganic ion flux [67]. Alternatively, a specific regulatory (inhibitory) receptor site may exist to which inhibitors bind before the channel opens and the signal is transmitted, thus affecting both k'_opening_ and k'_closing_. Indeed, the use of a caged carbachol allowed to determine the effect of the local anesthetic procaine on both k'_opening_ and k'_closing_, thereby corroborating the second hypothesized mechanism [67].

Hess’ caged carbachol **5** was used also by Chan and Evans to study the kinetics of activation of the Ca^2+^‐dependent K^+^ current induced in outer hair cells (OHCs), that in the mammalian cochlea receive a predominantly cholinergic efferent innervation from the brainstem [68]. The application of ACh activates a Ca^2+^‐dependent K^+^ current in OHCs, and the resulting hyperpolarization is an important part of the inhibition mediated by cholinergic efferent nerve fibers to the cochlea. Electrical stimulation of the efferent nerves produces a decrease in spike discharge rate in auditory nerve fibers. The OHC efferent synapse has an unusual pharmacology, being sensitive to both nicotinic and muscarinic antagonists, although—as more recently clarified—cochlear and vestibular hair cells only express functional α_9_α_10_ nAChRs [69]. To assess the contribution of the OHCs to the relatively slow kinetics of the electrically stimulated efferent response, the authors measured the kinetics of the cells’ response to carbachol released from its caged form by flash photolysis with UV light. A delay in the onset of the outward K^+^ current following photorelease of carbachol was consistently observed, and the activation phase of the response could be described by a sigmoidal‐like function with a mean delay of 59 ms and time constant of 71 ms. The sum of these values lies within the time scale reported for the onset of the inhibition following electrical stimulation of the efferent nerves. Although a distinct AChR current was not observed in these experiments, the authors concluded that the voltage‐sensitivity of the response and its dependence on external Ca^2+^ indicate that carbachol could trigger Ca^2+^ entry.

Another application of the same derivative **5** was implemented and published in 2003 by Yakel's group [70]. To contribute to the investigation of the physiological role of nAChRs in regulating neuronal excitability and their involvement in hippocampal plasticity, caged carbachol was used as a tool to map the distribution of receptors on the membrane of rat hippocampal CA1 stratum radiatum interneurons and pyramidal cells in acute slices by recording nAChR‐mediated currents elicited by local UV laser‐based photolysis in patch‐clamped neurons. They found that functional nAChR receptors are preferentially expressed on interneurons, but not on pyramidal cells, consistent with previous results [71]. α_7_‐containing nAChRs (later identified as homomeric α_7_ nAChRs) emerged as the predominant subtype expressed on these interneurons, located primarily at the soma and proximal dendrites. In addition, α_7_ nAChRs were found to be significantly Ca^2+^‐permeable and the recovery from desensitization was much faster in cells dialyzed with the Ca^2+^ chelator BAPTA. Overall, these results indicated a strategic location and Ca^2+^ regulation of α_7_ nAChRs, supporting their key function in hippocampal excitability and plasticity.

**Figure 3:**
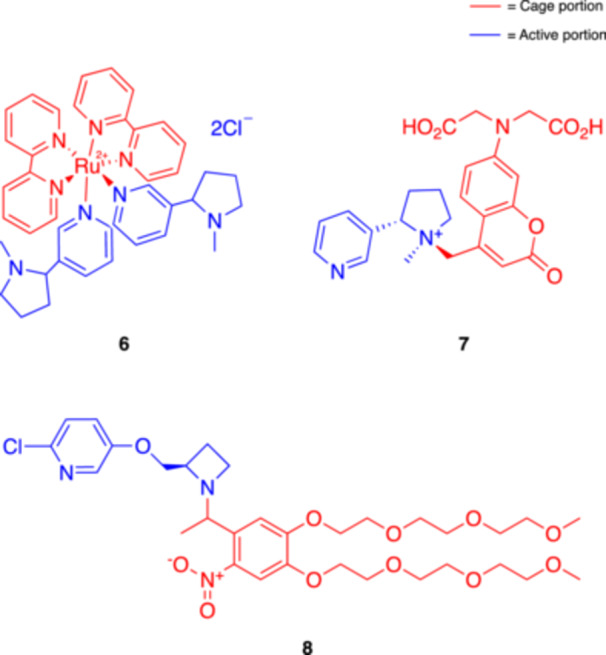
Photocleavable nicotine‐based (**6** and **7**) and ABT594‐based (**8**) caged agonists of nAChRs. [Color figure can be viewed at wileyonlinelibrary.com]

A few years later, the synthesis and characterization of the first caged nicotine was published by Etchenique and coll., based on the use of ruthenium(II) polypyridine complexes [42]. The caged nicotine complex ([Ru(bpy)_2_(Nic)_2_]^2+^), also known as RuBi‐Nicotine (RuBi‐Nic, **6**, Figure 3), could be photolyzed using blue (473 nm) or green (532 nm) light, leading to the fast release of nicotine at safe wavelengths. This compound showed good water solubility and stability under physiological conditions and was able to elicit action potentials after photolysis in leech neurons. RuBi‐Nic was also applied more recently in another study by Shuba and coll. to stimulate the proliferation of A549 lung cancer cells [72]. Photoreleased nicotine induced cell proliferation similarly to free nicotine. Importantly, the byproducts resulting from the photolysis of RuBi‐Nic proved to be non‐toxic, further supporting the safety of this method. Altogether, these two cases demonstrated the potential of RuBi‐Nic to function as a safe tool for studying the effects of nicotine with high spatiotemporal resolution.

Various neurotransmitters and pharmacological agents cannot be caged via standard strategies because they lack obvious attachment groups (e.g., COOH, OH, NH) for photolabile moieties. In this regard, in 2018 Lavis and coll. illustrated a novel strategy for “caging” tertiary amines by attaching a coumarin cage, thus forming a quaternary ammonium salt. As a proof of the concept, they applied the new method to generate a photoactivatable nicotine, named PA‐Nic (**7**, Figure 3) [43, 73]. PA‐Nic releases nicotine upon exposure to UV light, with a high extinction coefficient and good stability in the dark. The study demonstrated that PA‐Nic could evoke nicotinic currents in mouse brain slices, offering superior performance compared to previous compounds [42]. PA‐Nic could be activated using one‐ or two‐photon excitation (2PE), allowing precise spatial and temporal control in various experimental settings. This approach is potentially useful for further dissecting the role of nAChRs in different neuronal circuits, particularly to better understand nicotine dependence.

In a follow‐up study published in 2018 by the same research groups, PA‐Nic was used to investigate how nAChRs in ventral tegmental area (VTA) glutamate neurons influence excitatory transmission [74]. VTA glutamate neurons are integral components of reward circuitry but whether they are subject to cholinergic modulation was unknown. By using a combination of molecular, physiological and photostimulation techniques, the researchers determined that these neurons express functional heteromeric nAChRs. These receptors modulate local glutamate transmission and provide a cellular mechanism by which nicotine acts to impact the brain's reward circuitry. More specifically, regulation of glutamate in the VTA by nicotine depends upon β_2_ subunit‐containing nAChRs to influence mesolimbic pathways related to addiction and motivated behaviors.

In another work published in 2019, Drenan and coll. used PA‐Nic as a tool to explore the changes in the habenulo‐interpeduncular circuitry after chronic nicotine exposure (CNE) [75]. Nicotine uncaging revealed that the CNE significantly enhanced the function of nAChRs on the proximal axonal membranes of the medial habenula neurons. Moreover, in the interpeduncular nucleus, especially the rostral subnucleus, CNE resulted in a more robust depolarization response to nicotine uncaging near these neurons. These findings suggest that CNE profoundly alters nicotinic cholinergic signaling and enhances cell excitability within the habenulo‐interpeduncular circuitry, a pathway critical to nicotine dependence.

PA‐Nic was applied in another work published in 2022 by Drenan and Jin [76]. The caged nicotine was here used in combination with 2PE laser scanning microscopy to examine the morphology of α_7_ nAChR‐expressing neurons in the interpeduncular lateral subnucleus (IPL) [76]. The researchers found that IPL neurons are sparse, with modestly sized cell bodies and relatively low dendritic complexity, similar to what was previously observed in IPN neuron types. Unlike some other IPN neurons, IPL neurons lacked dendritic spines. Since only a fraction of IPL neurons showed Chrna7 mRNA expression and functional α_7_ responses, the authors suggested that α_7_ nAChR expression may identify a specific subset of IPL GABAergic neurons.

In parallel, the group of Ellis‐Davies developed a novel photocleavable nicotinic ligand based on ABT594 [44], a potent and selective α_4_β_2_ nAChR agonist by Abbott [77]. ABT594 bears a secondary amine functionality suitable for caging with a nitrobenzyl chromophore. The role of nAChRs is particularly critical in modulating neuronal communication in the medial habenula (MHb), a region implicated in nicotine addiction. Caged ABT594 (i.e., di‐polyethyleneglycol‐nitrobenzyl‐ABT594 **8**, Figure 3) was used for probing nAChRs in the MHb of the mouse brain. The authors used both one‐ and 2PE to photorelease ABT594 for the real‐time measurement of ion channel activity and Ca^2+^ transients in various parts of the neurons, including their somas, dendrites, and axons. Notably, the study demonstrated that these receptors are not only present in the cell bodies but also along the axons of MHb neurons, where they mediate large Ca^2+^ signals. Since axonal nAChRs had not been extensively investigated before, the results of this study suggested a novel role for these receptors in nicotine signaling. Given that the MHb is involved in nicotine withdrawal and reinforcement pathways, the results of this research offered new insights into the mechanisms modulating nicotine addiction and indications in view of putative treatments targeting these pathways.

### 3.2 Photoswitchable Ligands

**Figure 4:**
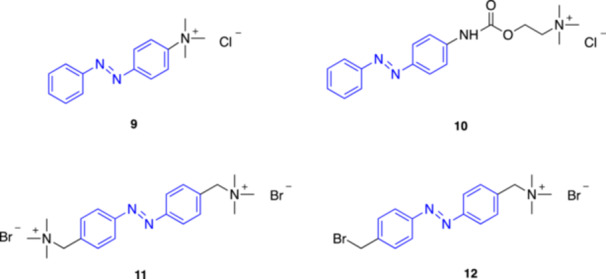
First discovered photoswitchable modulators of AChRs. [Color figure can be viewed at wileyonlinelibrary.com]

In 1969, Erlanger and Nachmansohn [45] demonstrated that the photoisomerizable compounds *p*‐phenylazophenyltrimethylammonium (azo‐PTA **9**, Figure 4) and N‐p‐phenylazophenyl‐*N*‐phenylcarbamylcholine (azo‐Ph‐carbachol **10**, Figure 4), derived respectively from the two known cholinergic agonists phenyltrimethylammonium and carbachol, act as photoswitchable inhibitors of acetylcholine receptors (AChRs). In particular, they both proved to function as antagonists in their *trans* state on the electroplax membrane of *Electrophorus electricus*, whereas exposure to 320 nm UV light (*trans*‐to‐*cis* isomerization) switched off the antagonistic activity of these molecules causing significant depolarization of the membrane in the presence of carbachol. At that time, it was unclear if this effect was due to nAChRs or mAChRs [78].

A couple of years later, Erlanger and Wassermann described BisQ (**11**, Figure 4), al photochromic nAChR agonist designed from the partial agonist decamethonium through an unusual azologization strategy [46], where an azobenzene unit replaced the carbon chain [47]. *Trans*‐BisQ proved to be a potent partial photoagonist of nAChRs (EC_50_ = 60–80 nM) on the electroplax membrane, causing significant depolarization that could be rapidly reversible under illumination at 360 nm light. Further studies revealed that, at low concentrations, the effect of *trans*‐BisQ can be blocked by the nAChR competitive antagonist tubocurarine. However, at increased *trans*‐BisQ concentration repolarization occurred even in the presence of tubocurarine, suggesting two distinct binding sites for *trans*‐BisQ, with only one overlapping with the tubocurarine binding site.

In parallel, Siliman and Karlin proposed to covalently attach an agonist to a nAChR via a thiol group artificially obtained after reduction of a disulfide bond in proximity of the receptor binding site [79]. Building on this, Erlanger and coll. designed QBr (**12**, Figure 4), an analog of BisQ in which one trimethylammonium group was replaced with a bromine [47]. This modification allows QBr to covalently tether to the receptor, enabling photocontrol of the distance of the agonist moiety from the binding pocket. Using this approach, no receptor desensitization was observed, unlike the strategy adopted by Karlin et al. Notably, both BisQ and tethered QBr are active in their *trans* configuration [80].

**Figure 5:**
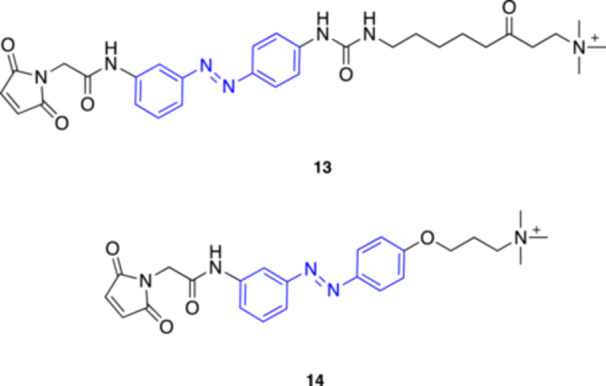
Photoswitchable modulators of nAChRs developed by Trauner's and Kramer's groups. [Color figure can be viewed at wileyonlinelibrary.com]

After a long quiet period in this field, in 2012 Trauner's and Kramer's groups introduced a pioneering approach for studying and manipulating nAChRs [48]. They combined synthetic chemistry with genetic engineering (optochemical genetics) to generate artificial light‐regulated nAChRs. To this end, they designed synthetic photoswitches that can be covalently bioconjugated to a reactive functional group (the sulfhydryl group of a cysteine) purposely introduced on the target receptor. The study focused on heteropentameric α_3_β_4_ and α_4_β_2_ nAChRs that were engineered with photoswitchable tethered ligands bearing a reactive maleimide tail. The engineered α_3_β_4_ and α_4_β_2_ nAChRs, expressed in *Xenopus* oocytes, were made light‐sensitive using the photoagonist MAACh (**13**, Figure 5). After conjugation, inward currents could be generated with 380 nm light, and the effect vanished at 500 nm. On the other hand, the covalent photoswitch MAHoCh (**14**, Figure 5) enabled light‐induced antagonism at 380 nm, partially blocking the effects of ACh, while strong inward currents were restored at 500 nm. Notably, the engineered receptors stayed functionally normal in the absence of light, ensuring their physiological role. Unlike traditional optogenetics, this approach requires minimal genetic manipulation, providing powerful new tools for investigating the intricate and nuanced functions of nAChRs in the brain [48].

In 2018, Mourot, Faure and coll. reported another innovative optochemical genetic approach to modulate nAChRs with light in the brain using freely moving mice [81]. Unlike traditional strategies, which generally aim to activate or inhibit specific neurons, they focused on interrupting neurotransmission at the post‐synaptic level, which should provide a more detailed understanding of how specific neurotransmitters and receptors modulate neural circuits and behaviors. They accomplished this by conjugating the photoswitchable covalent ligand MAHoCh to a cysteine‐mutated version of β_2_‐containing nAChRs in the VTA, a midbrain region controlling a wide range of behavioral repertoires, including reward processing. MAHoCh‐labeled receptors became photocontrollable while retaining their normal function in the dark. This allowed for the selective manipulation of VTA neurons, including dopamine (DA) neurons, without affecting nAChRs in other brain areas. Importantly, the authors showed that postsynaptic β_2_‐nAChR activation by endogenous ACh was able to modulate both tonic and burst firing modes of VTA DA neurons, while light‐promoted inhibition of β_2_‐nAChRs reduced nicotine‐induced reinforcement learning in mice, suggesting their critical function in nicotine addiction.

**Figure 6:**
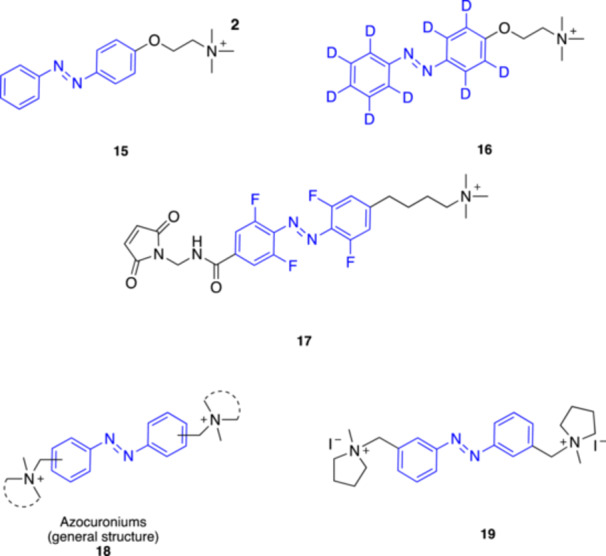
Most recently developed photoswitchable modulators of nAChRs. [Color figure can be viewed at wileyonlinelibrary.com]

In 2015 Trauner's group presented AzoCholine (**15**, Figure 6), a new freely diffusible photochromic ligand designed to control α_7_ nAChRs [49]. They showed that AzoCholine can be switched between *trans* and *cis* using 360 nm and 440 nm light, behaving as a potent α_7_ nAChR agonist (dark active) in electrophysiological studies in HEK293T cells. Its efficacy was confirmed in more complex biological systems, such as rat dorsal root ganglion neurons and *Caenorhabditis elegans*. In addition, the authors revisited BisQ [47], confirming its role as an agonist for muscle‐type nAChRs but not for neuronal α_7_ nAChRs.

Recently, Li and coll. published the results of a study that investigates the effects of deuteration in photopharmacology [50]. They developed a deuterated version of the α_7_ photoagonist AzoCholine using a D‐labeled azobenzene unit. The deuterated photo‐agonist, named d_9_‐AzoCholine (**16**, Figure 6), retained the majority of the photophysical properties of AzoCholine. Cytotoxicity tests in HEK293T cells showed no difference between the two ligands, supporting the safety of deuterium incorporation. Interestingly, compared to AzoCholine, d_9_‐AzoCholine displayed higher efficacy (in *trans*) and enhanced photopharmacological properties in Ca^2+^ flux experiments. The authors also compared the effects of the two analogs in *Caenorhabditis elegans*. While there was no difference between the two compounds under 365 nm light, d_9_‐AzoCholine showed a significantly stronger capacity to inhibit the thrashing movements of a specific mutant strain under 455 nm light. Overall, the deuteration of the photoisomerizable unit in AzoCholine improved its photopharmacological performance, confirming in general the potential of deuteration in photopharmacology, although the mechanism underlying this intriguing effect remains unclear.

Conventional azobenzene‐based photoswitches often suffer from UV light activation and rapid thermal relaxation of the unstable *cis* form. To overcome these limitations, Ellis‐Davies and co‐workers developed light‐regulated probes for nAChRs using the tetrafluoroazobenzene (4FAB) scaffold as molecular photoswitch [51]. 4FABs ensure exceptional thermodynamic stability in the *cis* conformation, with a half‐life exceeding 12 days at 37°C in physiological conditions, along with photostationary states (*cis*/*trans* isomers) greater than 84%. Additionally, the well‐separated n–π* absorption bands of the *trans*‐ and *cis*‐4FAB isomers enable efficient photoswitching using visible light (green and violet) in both directions. The first probe, named 4FABTA (**17**, Figure 6), was designed as a freely diffusible photoswitchable ligand containing the trimethylammonium (TA) head group, enabling it to function as a nAChR agonist. The probe activated nAChRs upon irradiation with violet light on mouse brain slices, and the effect disappeared under green light. Unexpectedly, attempts to conjugate a maleimide‐containing derivative of this probe with a cysteine on a genetically modified nAChR did not afford the desired light‐responsive channel. Despite this, the freely diffusible 4FABTA probe shows promise for interesting applications in neurobiology, particularly for controlling native nAChRs in vivo over extended periods and using visible light.

A novel class of photoswitchable neuromuscular blocking agents was introduced in 2020 by Rodríguez‐Franco and coll [52]. These ligands, termed “azocuroniums” (**18**, Figure 6), were based on azobenzene scaffolds linked to two *N*‐methyl‐*N*‐carbocyclic quaternary ammonium groups. Among the synthesized compounds, *meta*‐azocuroniums such as **19** (Figure 6) exhibited significant selectivity for muscle‐type over neuronal (α_7_ and α_4_β_2_) nAChRs, with minimal toxicity and no CNS penetration. Electrophysiological studies on *Xenopus laevis* oocytes confirmed that these compounds act as neuromuscular blockers, except for one derivative that behaves as an agonist. In general, the size and the hydrophobic character of the ammonium groups appeared to determine whether the azocuroniums would act as antagonists or agonists at the nAChRs. This class of molecules bear potential as muscle relaxants during surgery, with fewer side effects compared to conventional agents.

Beyond their biomedical significance, nAChRs are also key molecular targets in agrochemistry, particularly in the design of insecticides. Several widely used pesticides, such as neonicotinoids, selectively bind to insect nAChRs, causing overstimulation of the nervous system, paralysis, and ultimately insect death. These compounds have been extensively studied to enhance potency and selectivity, although concerns regarding their environmental impact have been raised. Although it falls outside the primary scope of our review, it is worth mentioning the rise of photopharmacology in this field as an innovative strategy to enhance selectivity and minimize environmental persistence [82]. For instance, Shao and coll. (2023) developed a photoswitchable agrochemical, dithienylethene‐imidacloprid (DitIMI), which enables optical control of invertebrate nicotinic nAChRs. DitIMI exhibited a dramatic shift in insecticidal activity upon UV‐light‐induced isomerization, with the closed‐form being remarkably more potent than the open‐form. This approach offers a precise, reversible method for modulating insect behavior and may enhance pesticide selectivity [83].

## 4 Muscarinic Acetylcholine Receptors

### 4.1 Photocleavable Ligands

Based on our literature findings, the first report about a photocleavable ligand applied to mAChRs was published in 2003 by Ashby, Tepikin, and co‐workers [84], who used for their scope the caged carbachol *N*‐[(α‐carboxy)‐2‐nitrobenzyl] carbamylcholine trifluoroacetate (Compound **5**, Figure 2a), a non‐selective caged agonist also used to investigate nAChR signaling (see above) [41]. The researchers employed this caged carbachol to precisely photoactivate mAChRs localized in the basal membrane of pancreatic acinar cells and examined receptor response through Ca^2+^ signaling without diffusional interference. Their results revealed that localized activation of basal receptors triggered global Ca^2+^ oscillations with initiation in the apical pole, independent of direct receptor‐channel interaction in that region. The study suggested that mAChR activation at the basal membrane communicates via intracellular signaling cascades with the apical region, where the Ca^2+^ release machinery is located, and highlighted the role of spatial compartmentalization in Ca^2+^ signaling offering insights into the complex regulation of pancreatic acinar cell function, which is crucial for understanding pathological conditions such as pancreatitis and other exocrine‐related diseases.

**Figure 7:**
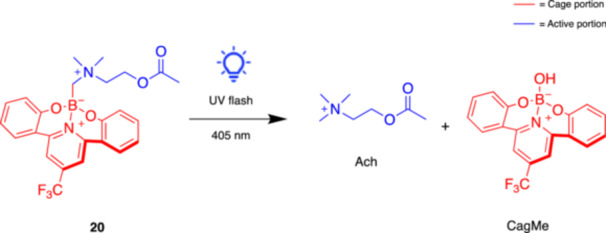
Photolytic reaction of **17** to release acetylcholine. [Color figure can be viewed at wileyonlinelibrary.com]

Recently, Ohmiya, Arai and coll. presented a novel radical caging strategy for cholinergic photopharmacology based on a photocleavable carbon‐boron (C–B) bond (Figure 7) [53], overcoming the limitations of traditional photocaging methods that require specific heteroatom‐based functional groups, thereby restricting their applicability. The authors developed a unique approach that cages bioactive molecules via carbon atoms, specifically utilizing carbon‐centered radicals generated through homolytic cleavage of C–B bonds under photoirradiation. As proof of the concept, the researchers reported the successful caging of ACh by installation of a CH_2_–B group on the nitrogen atom of ACh (CagMe‐ACh **20**, Figure 7). Uncaging could be achieved in a few seconds of irradiation with a 405 nm laser available in many commercial confocal microscopes, making this method very practical. The strategy was validated through experiments in HEK293T cells expressing a biosensor for ACh and Ca^2+^ imaging in *Drosophila* brain cells. The caged ACh probes remained stable in both air and aqueous environments and exhibited favorable uncaging kinetics with high efficiency. More in general, this study introduces a novel robust and versatile caging method that expands the toolkit for photopharmacology, potentially bringing back into play all those bioactive compounds and drugs previously excluded due to the absence of functional groups suitable for classical caging strategies.

### 4.2 Photoswitchable Ligands

The first report about a photoswitchable ligand active on mAChRs was published in 1982 by Erlanger, Wassermann and coll [85]. that used BisQ (**11**, Figure 4) as a tool to study mAChRs in the frog myocardium. They found that BisQ behaves also as a mAChR antagonist in the *trans* form, since it blocked the binding of [^3^H]‐*N*‐methylscopolamine and inhibited the K^+^ conductance triggered by muscarinic agonists, while the *cis* isomer resulted significantly less potent. Using voltage‐clamped frog atrial trabeculae to investigate the kinetics of the muscarinic‐dependent K^+^ conductance, the authors found that, upon exposure to carbachol and photoswitchable BisQ, the kinetics of conductance changes were temperature‐sensitive but largely independent of drug concentration, agonist type, or membrane voltage. This suggested that the rate‐limiting step in muscarinic‐dependent K^+^ conductance is not the ligand‐receptor binding but occurs in subsequent steps with high activation energy.

**Figure 8:**
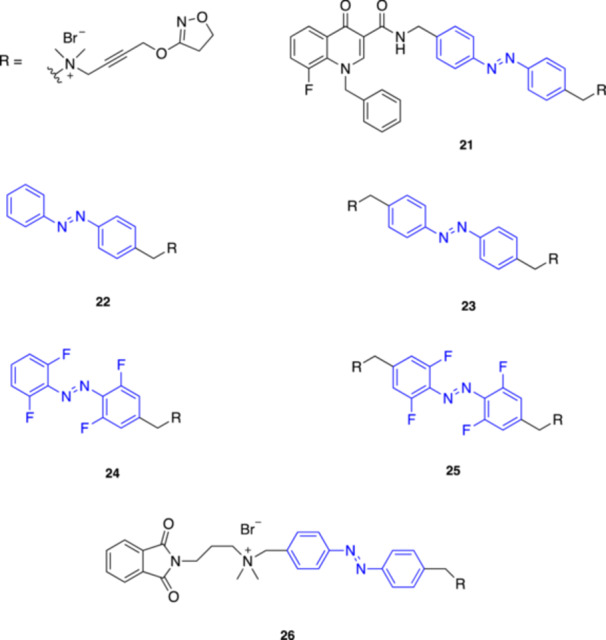
Iperoxo‐based photoswitchable modulators of mAChRs. [Color figure can be viewed at wileyonlinelibrary.com]

Several years later, Decker and co‐workers introduced BQCAAI (**21**, Figure 8), a novel photoswitchable compound targeting mAChRs, specifically the M_1_ subtype, which was also the first photoswitchable dualsteric ligand reported in the literature [54]. BQCAAI, inspired by a prior set of non‐photoswitchable dualsteric compounds [86], was designed to bind both the orthosteric site and an allosteric site on the M_1_ mAChR, with an azobenzene spacer, between the orthosteric and the allosteric pharmacophore, that enabled to photocontrol (at 360 and 460 nm) their distance and hence binding affinity and intrinsic efficacy of the dualsteric ligand. Fluorescence resonance energy transfer (FRET) studies in vitro confirmed that BQCAAI is a photoswitchable modulator of M_1_ mAChR displaying significant efficacy al low micromolar concentrations, behaving as an agonist in the *trans* state and an antagonist in the *cis* state. This compound represents an unprecedented tool for studying mAChR activation mechanisms and could lead to more targeted therapeutic approaches for diseases like AD, where selective M_1_ receptor activation would be beneficial.

In another study published in 2019, Decker and coll. presented their results about the effects of fluorination of the azobenzene core in a new set of photoswitchable M_1_ mAChR ligands [55], starting from the potent mAChR superagonist iperoxo as parent compound for their design [87, 88]. As illustrated in Figure 8, they generated photoswitchable monovalent (**22**) and bivalent (**23**) derivatives of iperoxo, as well as their red‐shifted variants (**24**, **25**) obtained by replacing the unsubstituted azobenzene with the tetra‐*ortho*‐fluorinated azobenzene. Both bivalent Compounds **23** and **25** exhibited significantly higher affinity than their corresponding monovalent analogues **22** and **24**, likely due to additional interactions with an allosteric binding site. As per design, the fluorinated variants **24** and **25** showed enhanced operational wavelengths, as they could be photoisomerized in both directions with visible light (500 nm for *trans*‐to‐*cis* and 400 nm for *cis*‐to‐*trans*, respectively), which is preferred for biological applications as it permits the use of less harmful light. Moreover, the fluorinated variants displayed also increased potency at the M_1_ mAChR. The bivalent fluorinated photoiperoxo **25** acted as an affinity switch, whereas the monovalent photoiperoxo **22** behaved as an efficacy switch. Overall, this work demonstrated that strategic fluorination of photoswitchable mAChR agonists can finely tune their photochemical as well as pharmacological properties, thereby enhancing their potential as tools for light‐controlled modulation of GPCR activity in vivo.

Riefolo, Matera, and coll. introduced the first photoswitchable M_2_ mAChR agonist, named Phthalimide‐Azo‐Iperoxo (PAI, **26**, Figure 8) [56], designed by replacing with an azobenzene unit the linker of a previously reported dualsteric agonist [89, 90]. PAI can be toggled between its *trans* and *cis* conformation by using 365 and 460 nm light. Ca^2+^ imaging assays in HEK cells confirmed that PAI is an M_2_ activator in the *trans* form, while illumination with UV light (*trans*‐to‐*cis* isomerization) remarkably reduced calcium oscillations. Intriguingly, PAI activated M_2_ mAChRs in the range of picomolar concentrations, preserving the potency of the parent superagonist iperoxo [88]. To validate the applicability of this method in vivo, the authors used PAI to control cardiac activity on Wistar rats. Intraperitoneal administration caused dose‐dependent bradycardia and PR interval lengthening, with stronger effects in the *trans* state at higher doses. However, the effects could not be reversed with light, likely due to insufficient tissue penetration, but they could be antagonized by atropine. To demonstrate reversible control of cardiac function in vivo, PAI was also applied to *Xenopus tropicalis* tadpoles. Administration of *trans*‐PAI directly to water readily reduced heart rate, in some cases causing cardiac arrest. UV light restored heart rate by converting PAI to its *cis* form, demonstrating remote and reversible heart rate control in vivo using light in non‐genetically modified, wildtype animals. In addition, the authors demonstrated with other Ca^2+^ imaging assays in HEK cells that PAI can be also activated using near‐infrared (NIR) light. PAI successfully induced Ca^2+^ responses under 2PE at 840 nm, achieving high potency at picomolar concentrations. Overall, PAI proved to be a powerful tool for precise, non‐invasive control of cardiac function for research and potential therapeutic applications, particularly in treating arrhythmias, where it may offer advantages compared to electric stimulation, which can create uneven areas of de‐ and hyper‐polarization, alter pH through faradaic reactions, and produce toxic gases.

In a follow‐up work taking advantage of M_2_ mAChR expression in the brain cortex, Barbero‐Castillo, Riefolo, and co‐workers used PAI to photocontrol brain state transitions [91]. The study, published in 2021, describes how PAI influences brain states by modulating the electrophysiological oscillatory activity patterns of the cortex both in vitro and in vivo through the light‐controlled activation of M_2_ mAChRs. First, the researchers investigated the effects of the non‐photoswitchable, non‐selective muscarinic superagonist iperoxo in cortical slices from ferrets, which intrinsically or during deep sleep/anesthesia exhibit slow oscillations (SOs). Iperoxo increased oscillatory frequency in a dose‐dependent manner but at higher concentrations caused hyperexcitability with seizure‐like discharges, which provided evidence that the capability of mAChR activation to alter cortical network dynamics requires receptor activation within physiological limits. Optically evoked PAI activation reduced the duration of the down states in SOs and enhanced the oscillatory frequency in both cortical slices and anesthetized mice. These findings show that PAI can reversibly and precisely control cortical rhythms, pointing to a new strategy in the study and possible treatment of disorders related to dysfunctional neural oscillations.

Two years later, Sortino and co‐workers reported another advancement in the fields of photopharmacology and neurobiology by demonstrating the application of three‐photon excitation (3PE) to control neuronal activity in vivo. 3PE enables even deeper penetration and higher resolution than 2PE, which are critical for studying deeply seated, complex neural circuits in three dimensions. For this purpose, they used PAI (**26**) to activate M_2_ mAChRs with 1560 nm (mid‐infrared) light [92], the longest wavelength reported so far for photoactivation in photopharmacology, both in vitro (HEK cells) and in vivo (zebrafish larvae). This work opens new avenues for non‐invasive and highly precise manipulation of brain activity in vivo in wildtype animals, which could significantly impact on basic neuroscience research and therapeutic interventions.

**Figure 9:**
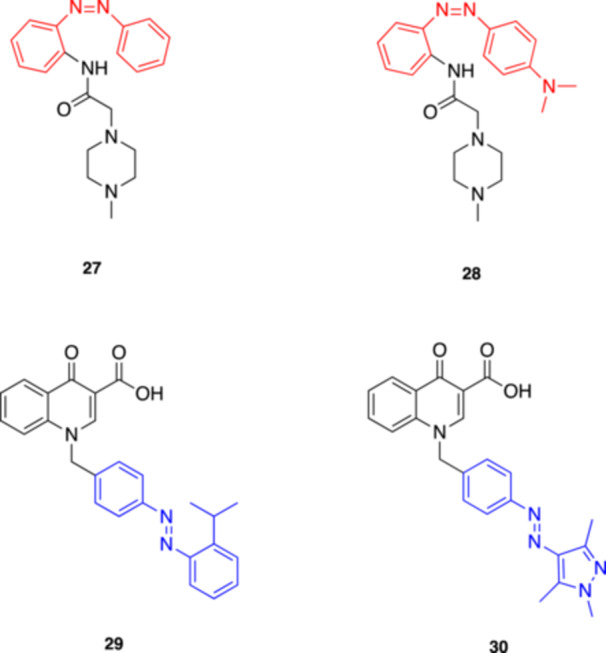
Most recently developed photoswitchable modulators of mAChRs. [Color figure can be viewed at wileyonlinelibrary.com]

In 2021 Riefolo et al. [57] presented another set of photoswitchable muscarinic ligands designed as unconventional analogs of tricyclic drugs, introducing the concept of “crypto‐azologization”, a novel design strategy to rationally convert tricyclic structures into photoisomerizable derivatives. As a testing ground for this new strategy, the authors drew inspiration from the mAChR antagonist pirenzepine to develop two photoisomerizable analogues, named cryptozepines (**27** and **28**, Figure 9). Both compounds proved to behave as antagonists in HEK cells expressing M_1_ mAChRs, with **27** showing stronger inhibition and maintaining the M_1_ selectivity of the parent compound. The effects of **27** were then tested in a more physiologically relevant setting, such as isolated mouse atria, where *cis*‐**27** was able to partially restore the heart rate initially decreased with carbachol, indicating M_1_ antagonism, while the *trans* isomer resulted inactive. This novel strategy broadens the azologization protocol to include tricyclic motif‐containing drugs where the photochromic scaffold is initially “hidden” and must be revealed through additional structural modifications beyond the standard azosteric replacement, potentially expanding the scope of photopharmacology and its applications to other targets of tricyclic drugs, e.g., serotonin and norepinephrine transporters.

Recently, Gerwe and coll. introduced the first photoswitchable positive allosteric modulator (PAM) targeting Class A GPCRs [58]. As known, GPCRs are prominent drug targets but pose challenges in selective modulation due to their high sequence conservation across receptor subtypes. The authors proposed the development of photoswitchable analogs of benzyl quinolone carboxylic acid (BQCA), an M_1_ mAChR PAM, to overcome these challenges. The new derivatives, termed photo‐BQCisA (**29**) and photo‐BQCtrAns (**30**) (Figure 9), incorporate an azobenzene moiety into the BQCA scaffold. The photodependent activity of the molecules was demonstrated in functional assays in which photo‐BQCisA exhibited PAM activity when irradiated with UV light (365 nm), while photo‐BQCtrAns could be deactivated using blue light (470 nm). The precision offered by these photoswitchable PAMs holds promise for both fundamental research and putative therapeutic applications for challenging targets like M_1_ mAChRs and other GPCRs on a broader scale.

## 5 Cholinesterases

### 5.1 Photocleavable Inhibitors and Reactivators

**Figure 10:**
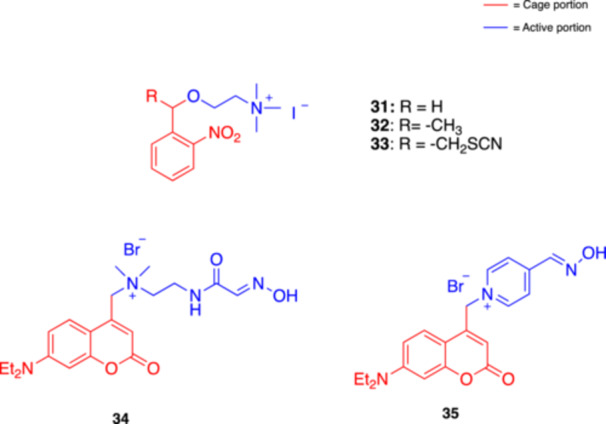
Photocleavable cholinesterase (AChE) modulators. [Color figure can be viewed at wileyonlinelibrary.com]

Based on our literature research, the first photocleavable compounds targeting cholinesterases were described in 1996 by Peng and Goeldner [59], that synthesized and characterized three photolabile choline precursors **31–33** (Figure 10) as reversible inhibitors of these enzymes. When exposed to light, these compounds released choline in a highly controlled manner and demonstrated different efficiencies, with derivative **31** showing the highest quantum yield and fastest photolysis rate. Using UV spectroscopy, HPLC analysis, and enzymatic assays, the researchers measured the kinetics of choline release and confirmed that the compounds effectively inhibited both AChE and BuChE. In a parallel study published in the same year, Goeldner and coll. further characterized **32** [59] and two other photolabile precursors of carbachol, indicated in this review as **2** and **5** (see Figure 2) and previously described by other authors [40, 41], as potential tools for time‐resolved crystallographic studies of cholinesterases [93]. Both **2** and **32** behaved as effective photoactivated inhibitors of AChE and BuChE, with inhibition constants in the micromolar range, rapid photolysis, and high quantum yield. Upon photolysis, the choline derivative regenerated AChE activity, while the carbamylcholine derivative led to a time‐dependent inactivation due to carbamylation of AChE. The latter could be reversed by dilution, which allowed decarbamylation of the enzyme. These photolabile compounds could serve as effective tools for probing the dynamics of cholinesterases in real‐time crystallography, allowing for synchronized initiation of enzymatic reactions within the crystal, thus facilitating studies of how products like choline exit the active site. Several years later, in 2022, Choi and coll. applied the photo‐uncaging approach to AChE reactivation. AChE inhibition by organophosphates (OPs), such as those found in nerve agents and pesticides, can lead to severe neurological damage or even death. The authors developed two coumarin‐caged oxime reactivators (**34** and **35**, Figure 10) and measured their biochemical activities in AChE assays in vitro [60]. Remarkably, caged oxime **34**, upon irradiation at 365 nm or 420 nm, successfully uncaged and released the oxime and was able to reactivate AChE, whereas no significant reactivation was observed in the dark. This work introduced a new method for precise reactivation of AChE that might lead to safer treatments against OP poisoning and provided a new tool for research in neurobiology and toxicology.

### 5.2 Photoswitchable Inhibitors

The first study on the optical control of AChE function was published in 1969 by Erlanger and coll. using azo‐PTA (**9**, see Figure 4) [94]. The authors found that this compound was most effective as AChE inhibitor in its *trans* form, while UV light‐induced isomerization to *cis* modestly decreased its activity. Soon after their first report, Erlanger and coll. described another photoswitchable AChE inhibitor, named azo‐carbachol (**10**, see Figure 4) [95]. Also in this case the *trans* isomer was identified as the most potent AChE inhibitor, while it produced only moderate changes in AChE activity upon isomerization to *cis* at 366 nm.

The pioneering work by Erlanger and his team was expanded several years later, in 2014, when Decker's and Trauner's groups independently introduced novel photoswitchable inhibitors of AChE. Both groups utilized tacrine (THA), an AChE inhibitor once used in the AD therapy, as the active pharmacophore. The main distinction between their approaches lays in the type of photoswitch that each group employed. Drawing inspiration from a set of molecules developed earlier [96], Decker and co‐workers designed their photoswitchable AChE inhibitor by linking two tacrine molecules with a 1,2‐dithienylethene (DTE) photoswitch (**36**, Figure 10) [61]. This DTE‐THA derivative can be converted to its closed form under UV light (312 nm), while the reverse isomerization to the open form is obtained at longer wavelengths (> 420 nm). The researchers determined an IC_50_ value of ∼19 nM for the closed form and ∼50 nM for the open one using the Ellman's assay. Notably, the achievable number of switching cycles was limited to eight by photobleaching, which restricts the use of this compound as a reversible AChE inhibitor. Trauner's group developed a photoswitchable tacrine derivative called azo‐THA (**37**, Figure 10), which was connected to an azobenzene photoswitch through the amine group of tacrine [62]. Azo‐THA inhibited AChE in its dark‐adapted form with a *K*_i_ of approximately 100 nM and in a photodependent way, reducing AChE activity to 17% under blue light (440 nm) and almost abolishing it completely (4%) under UV light (350 nm), and this behavior remained stable over multiple light cycles. Additional experiments on mouse trachea tissue demonstrated the induction of photodependent relaxation in tracheal muscles, which contract in response to ACh. *Cis*‐azo‐THA (stronger AChE inhibition) led to slower muscle relaxation (∼15 s), while application of *trans*‐azo‐THA resulted in faster muscle relaxation (∼11 s). Overall, the authors demonstrated that azo‐THA acts as a reversible photochromic inhibitor of AChE both in vitro and ex vivo, and could be used as a photopharmacological tool to control synaptic transmission at the neuromuscular endplate through light‐dependent regulation of neurotransmitter clearance.

In 2022, Decker, Maurice and coll. described the development of photoswitchable inhibitors for BuChE [63]. They synthesized several derivatives by incorporating the azobenzene moiety into the structure of existing inhibitors, using both azologization and azo‐extension as design strategies. *In vitro* experiments showed that these compounds behave as highly selective BuChE inhibitors over AChE, with two derivatives, **38** and **39** (Figure 10), displaying respectively a 6‐ and 10‐fold difference in the IC_50_ values between their photoisomers. In a mouse model of AD, Compound **39** was able to restore cognitive function upon illumination, underscoring its potential for non‐invasive, light‐controlled therapy. Although further development is certainly required for non‐invasive in vivo photoswitching to enhance the therapeutic potential of such compounds, this research opens new possibilities for precise and reversible treatment strategies in neurodegenerative diseases like AD.

**Figure 11:**
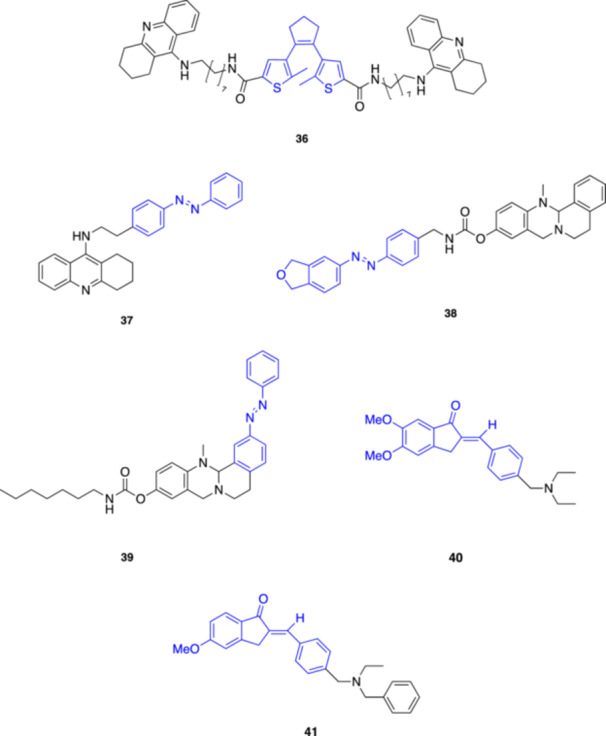
Photoswitchable cholinesterase modulators. [Color figure can be viewed at wileyonlinelibrary.com]

Recently, the groups of Altomare and Corelli further expanded the toolbox of light‐controlled cholinesterase inhibitors by integrating photopharmacology with the multitarget‐directed ligand design strategy. In a first study published in 2022 they developed a small library of cinnamic acid‐inspired photoswitchable multitarget inhibitors targeting both AChE and monoamine oxidase B (MAO‐B), both implicated in neurodegenerative diseases [64]. These compounds demonstrated high selectivity for AChE and MAO‐B over BuChE and MAO‐A, coupled with remarkable differences in the inhibition potency between the two isomers. Notably, a 5‐methoxyindan‐1‐one derivative (**40**, Figure 10) exhibited the most promising biological activity, demonstrating strong inhibitory effects in the submicromolar range against both enzymes (IC_50_ values of 0.11 μM for AChE and 0.26 μM for MAO‐B). Based on these findings, the authors further expanded the series of 5‐methoxyindan‐1‐ones by developing new congeners where the tertiary amino group of **40** was modified to include alkyl groups with varying degrees of bulkiness and lipophilicity [65]. All compounds exhibited in their *trans* state potent inhibition of AChE, with submicromolar IC_50_ values and good selectivity over BuChE. Among them, Compound **41** (Figure 10), bearing an *N*‐benzyl(ethyl)amino group, acted as the most potent AChE inhibitor, with an IC_50_ value of 39 nM, threefold stronger than its parent Compound **40** in the *trans* conformation. However, its activity decreased only slightly upon UV‐B irradiation. All compounds in *trans* also showed submicromolar inhibition of MAO‐B, with minimal variation upon photoisomerization. Overall, these studies provide evidence of the potential of this new class of photoswitchable compounds, able to synergistically inhibit the two neurodegenerative disease‐related enzymes AChE and MAO‐B, with the added value given by the possibility of photomodulating their pharmacological effect. Clearly, poor tissue penetration and the potential cytotoxicity of UV radiation strongly limit any in vivo applications of such molecules, thus requiring additional efforts to approach the final goal, like using 2PE of the developed compounds or introducing red‐shifted photoswitchable groups (Figure 11).

## 6 Final Remarks

The application of photopharmacology to cholinergic targets (membrane receptors and enzymes) has significantly deepened our understanding of cholinergic signaling, an essential actor in the nervous system responsible for numerous physiological functions, including cognitive processes, muscle activation, and autonomic regulations, thereby opening new perspectives for therapeutic interventions in neurological disorders. In this review we examined the progress made in the last decades in modulating cholinergic signaling using photoswitchable and photocleavable ligands.

Photoswitchable ligands enable the reversible control of receptor activity by toggling between inactive and active states upon exposure to specific wavelengths. For example, azobenzene‐based ligands have been successfully used to modulate nAChRs and offer a non‐invasive way to investigate receptor dynamics at high temporal resolution. On the other hand, photocleavable ligands undergo irreversible bond cleavage upon light exposure and thus provide a means to permanently activate or deactivate cholinergic pathways. This has been particularly useful in mapping cholinergic circuits in the brain, where spatially and temporally precise modulation is essential. Moreover, the combination of photopharmacology with advanced imaging techniques has enabled real‐time monitoring of cholinergic activity, facilitating the discovery of novel therapeutic targets.

Despite the progress made, several challenges continue to stand in the way of fully exploiting the potential of photopharmacology. Among the major limitations, poor penetration of short‐wavelength light in biological tissues severely limits the in vivo efficacy of this approach. UV and blue light, typically used in photopharmacology for photoisomerization or for photo‐uncaging, are rapidly absorbed or scattered by biological tissues, resulting in poor penetration and limited applicability in deeper organs and tissues, such as the brain. In addition, prolonged exposure to these wavelengths can be phototoxic, leading to cellular damage, which further hinders the clinical translation of these methods. Advances in synthetic chemistry, molecular biology and photonics will be critical in overcoming such limitations and expanding the utility of photopharmacological tools. There is increasing emphasis on developing ligands and methods that utilize red or IR light, which penetrate biological tissues more effectively, are less harmful, and allow three‐dimensional focalization like 2PE and 3PE. Red‐shifted chromophores, for instance, have been engineered to absorb light in the red or near IR spectrum, offering a promising solution for deeper tissue photopharmacology [97]. Additionally, upconversion nanoparticles, which transform lower‐energy NIR light into higher‐energy UV or visible light, have been explored as a method to enable deeper tissue activation of traditional light‐responsive ligands [98]. Multiphoton activation also holds promise by utilizing simultaneous absorption of two or more photons to achieve ligand activation with longer wavelengths [92, 99]. Multiphoton microscopes are becoming widely available in research facilities and allow in some cases to use the previously reported UV‐photoswitching compounds.

As all these technologies evolve, they could 1 day change how many disorders are treated, offering new hope for therapies that are both highly precise and effective. Overall, the outlook for cholinergic photopharmacology is bright, with new perspectives for innovative precision medicine, especially in neurodegenerative diseases. Light‐regulated noninvasive (and possibly reversible) methods for controlling cholinergic signaling pathways will enable the modulation of specific neuronal circuits with unprecedented accuracy. For instance, targeted modulation of muscarinic and nicotinic acetylcholine receptor subtypes could potentially ameliorate cognitive deficits associated with AD by restoring cholinergic function in affected brain regions.

## Conflicts of Interest

The authors declare no conflicts of interest.

## Data Availability

The authors have nothing to report.
